# SlBBX20 interacts with the COP9 signalosome subunit SlCSN5-2 to regulate anthocyanin biosynthesis by activating *SlDFR* expression in tomato

**DOI:** 10.1038/s41438-021-00595-y

**Published:** 2021-07-01

**Authors:** Dan Luo, Cheng Xiong, Aihua Lin, Chunli Zhang, Wenhui Sun, Junhong Zhang, Changxian Yang, Yongen Lu, Hanxia Li, Zhibiao Ye, Ping He, Taotao Wang

**Affiliations:** 1 Key Laboratory of Horticulture Plant Biology, Ministry of Education, Huazhong Agriculture University, 430070 Wuhan, China; 2 Department of Biochemistry and Biophysics, Texas A&M University, College Station, TX 77843 USA

**Keywords:** Plant molecular biology, Molecular engineering in plants

## Abstract

Anthocyanins play vital roles in plant stress tolerance and growth regulation. Previously, we reported that the photomorphogenesis-related transcription factor SlBBX20 regulates anthocyanin accumulation in tomato. However, the underlying mechanism remains unclear. Here, we showed that SlBBX20 promotes anthocyanin biosynthesis by binding the promoter of the anthocyanin biosynthesis gene *SlDFR*, suggesting that SlBBX20 directly activates anthocyanin biosynthesis genes. Furthermore, we found by yeast two-hybrid screening that SlBBX20 interacts with the COP9 signalosome subunit SlCSN5-2, and the interaction was confirmed by bimolecular fluorescence complementation and coimmunoprecipitation assays. *SlCSN5* gene silencing led to anthocyanin hyperaccumulation in the transgenic tomato calli and shoots, and *SlCSN5-2* overexpression decreased anthocyanin accumulation, suggesting thSlCSN5-2 enhanced the ubiquitination of SlBBX20 and promoted the degradation of SlBBX20 in vivo. Consistently, silencing the *SlCSN5-2* homolog in tobacco significantly increased the accumulation of the SlBBX20 protein. Since SlBBX20 is a vital regulator of photomorphogenesis, the SlBBX20-SlCSN5-2 module may represent a novel regulatory pathway in light-induced anthocyanin biosynthesis.

## Introduction

Anthocyanins are pigments synthesized by the flavonoid pathway involved in the coloring of various organs, such as leaves, fruits, and flowers^1,2^. Anthocyanins accumulate in response to plant hormones, low temperature, high temperature, strong light, UV-B radiation, and other environmental factors^2–8^. Moreover, fruit color is a significant indicator of quality. Anthocyanins are the main pigments responsible for determining color in a broad variety of fruits. Anthocyanins also play significant roles as antioxidants in plant biotic and abiotic stress tolerance and thereby facilitate plant resistance to pathogens and insects.

*Aft* and *Atv* are two important loci that regulate anthocyanin biosynthesis in tomato. The *Aft* gene from tomato was mapped to chromosome 10 and found to encode a SlAN2-like R2R3-MYB protein that promotes anthocyanin biosynthesis^9,10^. *Atv* is located on chromosome 7 and encodes the SlMYBATV protein, which negatively regulates the synthesis of anthocyanins. In addition to positively regulating anthocyanin biosynthesis, Aft was reported to directly activate *SlMYBATV* expression. Additionally, SlMYBATV competes with Aft for interaction with the transcription factor SlJAF13, thereby downregulating the accumulation of anthocyanins in tomato fruit. Mutation of *SlMYBATV* results in the release of SlJAF13, which interacts with Aft, further leading to the upregulation of *SlAN1* and *SlAN11* expression and accumulation of anthocyanins in tomato fruit^9,11^.

Anthocyanin accumulation is mostly regulated by transcription factors and structural genes, including *CHS, CHI, F3H, F3’H, F3’5’H, DFR, ANS,* and *UFGT*^8,12^. The biosynthesis of anthocyanins is regulated by different transcription factors. The MYB-bHLH-WD40 (MBW) complex plays vital roles in regulating the biosynthesis of anthocyanins. The molecular mechanism by which the MBW complex regulates anthocyanin biosynthesis has been extensively studied. The WD40 protein likely plays a more general role in the regulation of the complex^8,13^. The activation of particular genes is determined by the expression pattern and DNA-binding specificity of the MYB and bHLH proteins.

*SlAN2* was reported to promote anthocyanin biosynthesis when plants were grown in strong light and under low-temperature conditions^14^. A recent study showed that the overexpression of *SlMYB75* induced the accumulation of anthocyanins^15^. Overexpression of *SlANT1* was reported to increase the expression levels of structural genes in the anthocyanin biosynthesis pathway^16^. The MdMYB1/10 gene was found to be involved in regulating the accumulation of anthocyanins in apple^17,18^. The MYB-TF gene *Cs6g17570* was identified as a vital player in the regulation of anthocyanin biosynthesis in blood oranges^19^. *MdMYB16* and *MdbHLH33* were also reported to be involved in anthocyanin metabolism^20^. In addition, MdbHLH3 was found to promote anthocyanin accumulation and fruit coloring under low-temperature conditions in apple^21^. The WD40 protein MdTTG1 was reported to interact with bHLH to regulate anthocyanin synthesis in apple^22^.

B-box (BBX) proteins are a class of zinc finger protein transcription factors that contain one or two B-box domains. Many studies in *Arabidopsis* have revealed that BBX family proteins play an important role in photomorphogenesis^23^. This particular group of BBX factors includes AtBBX1, AtBBX4, AtBBX19, AtBBX20, AtBBX21, AtBBX22, AtBBX23, AtBBX24, AtBBX25, AtBBX28, AtBBX30, AtBBX31 and AtBBX32^24–31^. The accumulation of anthocyanin is a general phenomenon in photomorphogenesis, and BBX proteins were also found to regulate anthocyanin synthesis. In pear, the BBX proteins PpBBX16, PpBBX18, PpBBX21, and PpBBX24 are involved in anthocyanin accumulation^5,6,32^. In apple, MdCOL4, MdBBX20, MdBBX22, and MdBBX37 were found to participate in the regulation of anthocyanin accumulation^2–4,33^.

In a previous study, we found that the SlBBX20 protein is modified by the CRL4 E3 ubiquitin ligase to regulate the biosynthesis of carotenoids in tomato fruit^34^. In addition to the carotenoid content, we found that overexpression of the *SlBBX20* gene led to a significant increase in the anthocyanin content. Here, SlBBX20 was found to target the *DFR* promoter and activate its expression. To further uncover the mechanism by which anthocyanin biosynthesis is regulated, we screened a yeast two-hybrid library and found that SlCSN5-2 interacts with SlBBX20. The downregulation of SlCSN5-2 resulted in the accumulation of anthocyanin in tomato. Furthermore, when we interfered with the expression of a *SlCSN5-2* homolog in tobacco, the expression level of the SlBBX20 protein was significantly increased, indicating that SlCSN5-2 regulates the accumulation of the SlBBX20 protein.

## Results

### Overexpression of *SlBBX20* led to increased anthocyanin accumulation

**Fig. 1:**
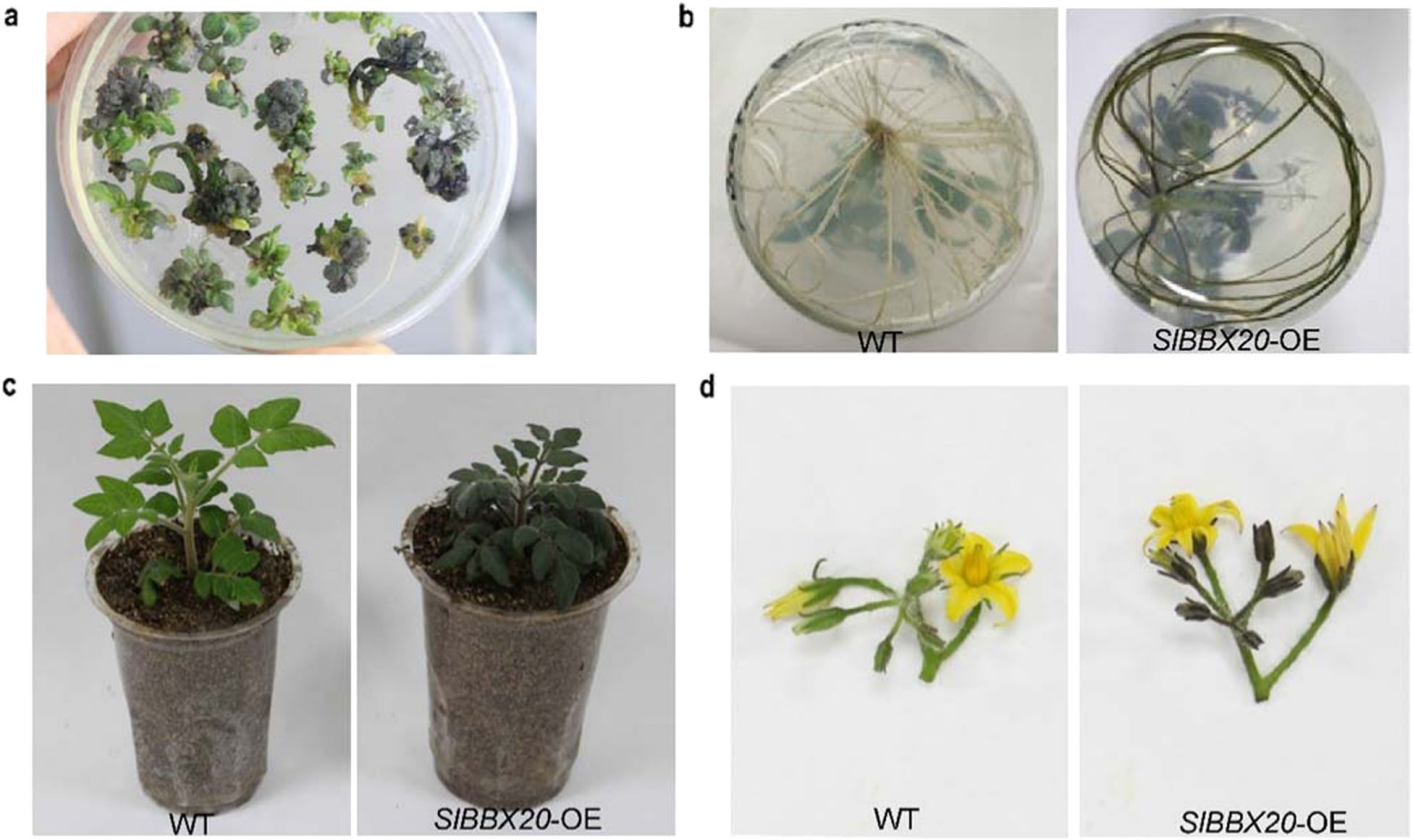
Anthocyanin accumulation in *SlBBX20*-overexpressing tomato lines. *SlBBX20* overexpression enhanced anthocyanin biosynthesis in the calli and shoots (**a**), roots (**b**), seedlings (**c**), and flowers (**d**) compared with that in the wild-type (WT) plants

To study the role of *SlBBX20* in regulating the accumulation of anthocyanins, we overexpressed full-length *SlBBX20* in tomato with the 35S promoter. In the transformation process, we found that the partial calli growing on the medium had become purple (Fig. 1a). After rooting, the seedlings and roots of the *SlBBX20-*overexpressing plants were also purple (Fig. 1b, c). Purple sepals in the flowers of *SlBBX20*-overexpressing plants were also observed (Fig. 1d). Thus, anthocyanins accumulated in diverse tissues with the overexpression of *SlBBX20*.

We also obtained three homozygous *SlBBX20-*knockout plants using CRISPR-Cas9 technology. No obvious difference in anthocyanin content was observed between the knockout plants and control plants, which may be due to redundant gene functions. Recent studies have shown that *Arabidopsis BBX20, 21* and *22* are functionally redundant and regulate hypocotyl elongation and anthocyanin accumulation as rate-limiting cofactors of HY5^35^. Therefore, we selected *SlBBX20*-overexpressing plants to perform follow-up experiments.

**Fig. 2:**
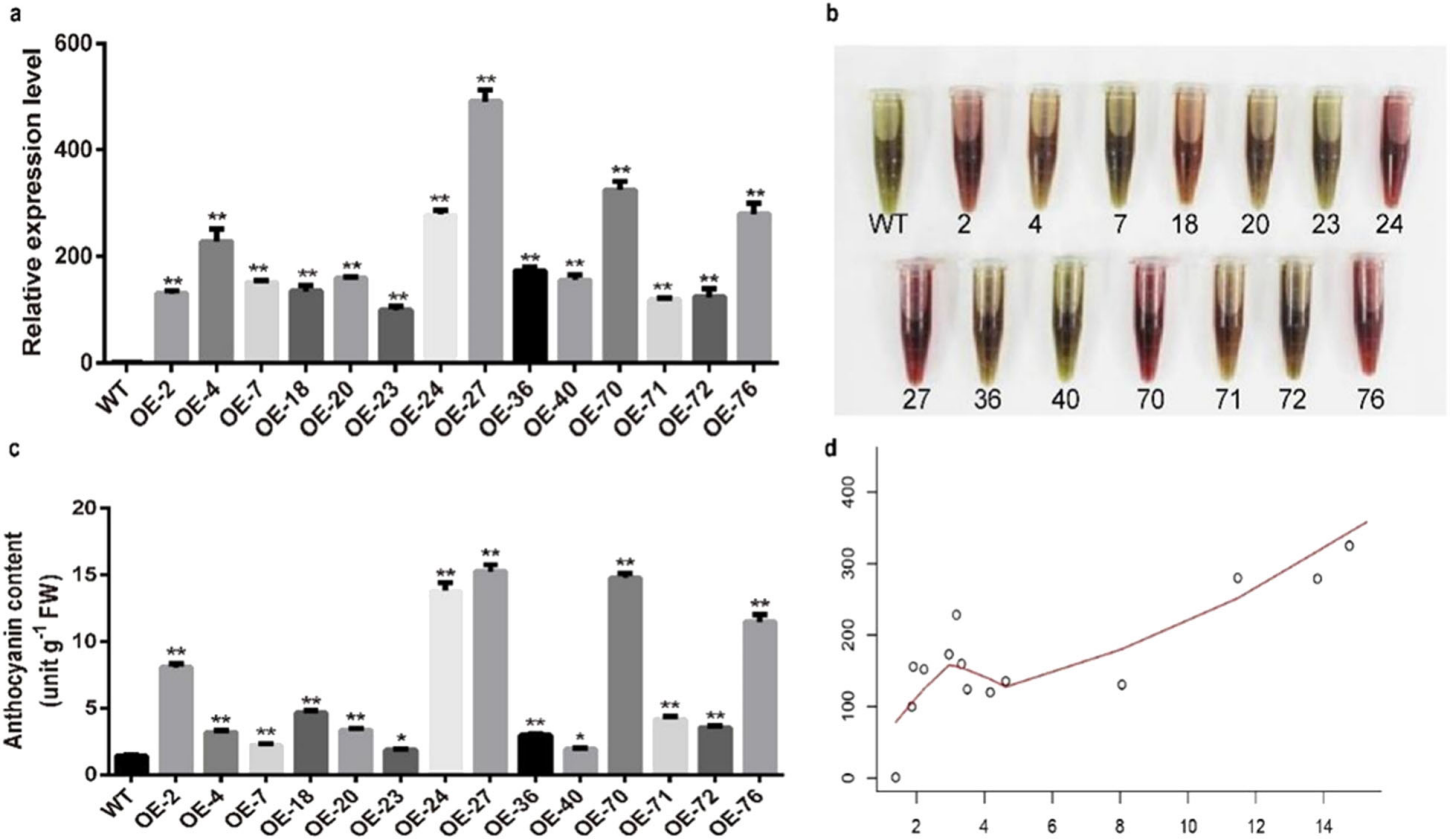
The correlation between anthocyanin content and *SlBBX20* expression in tomato. **a** Relative *SlBBX20* expression levels in different *SlBBX20*-overexpressing lines. **b** Extracts containing anthocyanins derived from the leaves of the corresponding *SlBBX20*-overexpressing lines. **c** Anthocyanin content in the leaves of the corresponding *SlBBX20*-overexpressing lines. **d** Scatter plot showing the correlation between relative *SlBBX20* expression and anthocyanin content. The bars show the mean ± SE (*n* = 3). A One-way ANOVA and Dunnett’s test were conducted. “*” and “**” indicate statistically significant differences with *P* < 0.05 and *P* < 0.01, respectively

We quantified the expression of *SlBBX20* and measured the anthocyanin content in 14 independent transgenic lines (Fig. 2a–c). The level of *SlBBX20* transcription was highly correlated with anthocyanin content. We further used the Pearson correlation coefficient to assess the correlation between these two parameters (Fig. 2d). The scatter plot showed the Pearson correlation coefficient between the relative *SlBBX20* expression level and level of accumulated anthocyanin to be 0.84, indicating a strong positive correlation between them.

### The expression of flavonoid biosynthesis genes was upregulated in *SlBBX20-*overexpressing lines

**Fig. 3:**
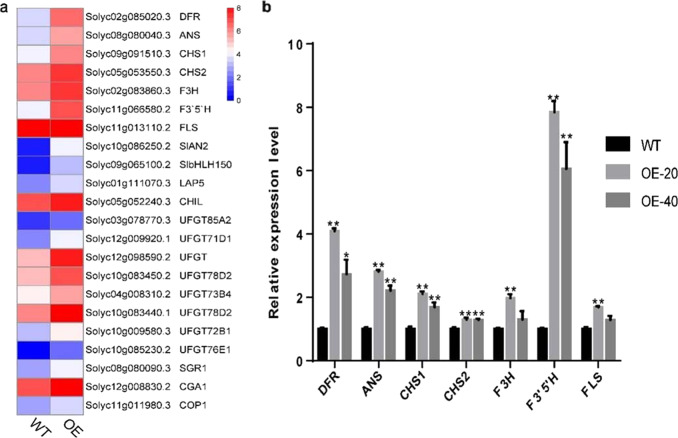
Expression of genes associated with pigment biosynthesis obtained from transcriptome data. **a** Heatmap of pigment-associated genes with increased expression in *SlBBX20*-overexpressing lines identified from an analysis of transcriptome data. **b** Validation of the transcriptome data using qRT-PCR. We validated the expression levels of structural genes associated with anthocyanin biosynthesis that were classified as upregulated based on an analysis of our transcriptome data

To explore the molecular mechanism by which *SlBBX20* regulates anthocyanin accumulation, we analyzed the transcriptomes of the *SlBBX20*-overexpressing plants and controls by RNA sequencing (RNA-Seq). Anthocyanins are synthesized by the flavonoid pathway. The expression levels of some genes in the pigment, flavonoid and anthocyanin biosynthesis pathways (*DFR, ANS, CHS1, CHS2, F3H, F3'5’H,* and *FLS*) were found to be upregulated in the *SlBBX20*-overexpressing plants (Fig. 3a). To independently validate these results, we quantified the expression of these anthocyanin-related structural genes in two *SlBBX20-*overexpressing lines, OE-20 and OE-40 (Fig. 3b). The expression levels of *CHS1*, *CHS2*, *F3H*, *F3'5’H, DFR,* and *FLS* were found to be upregulated in the *SlBBX20-*overexpressing lines relative to the control line. Among these genes, *DFR, ANS,* and *F3'5’H* were elevated more than twofold. This result is consistent with the transcriptome data, and these data imply that *SlBBX20* promotes the accumulation of anthocyanins by regulating the expression of anthocyanin biosynthesis genes.

### SlBBX20 directly regulates *SlDFR*

**Fig. 4:**
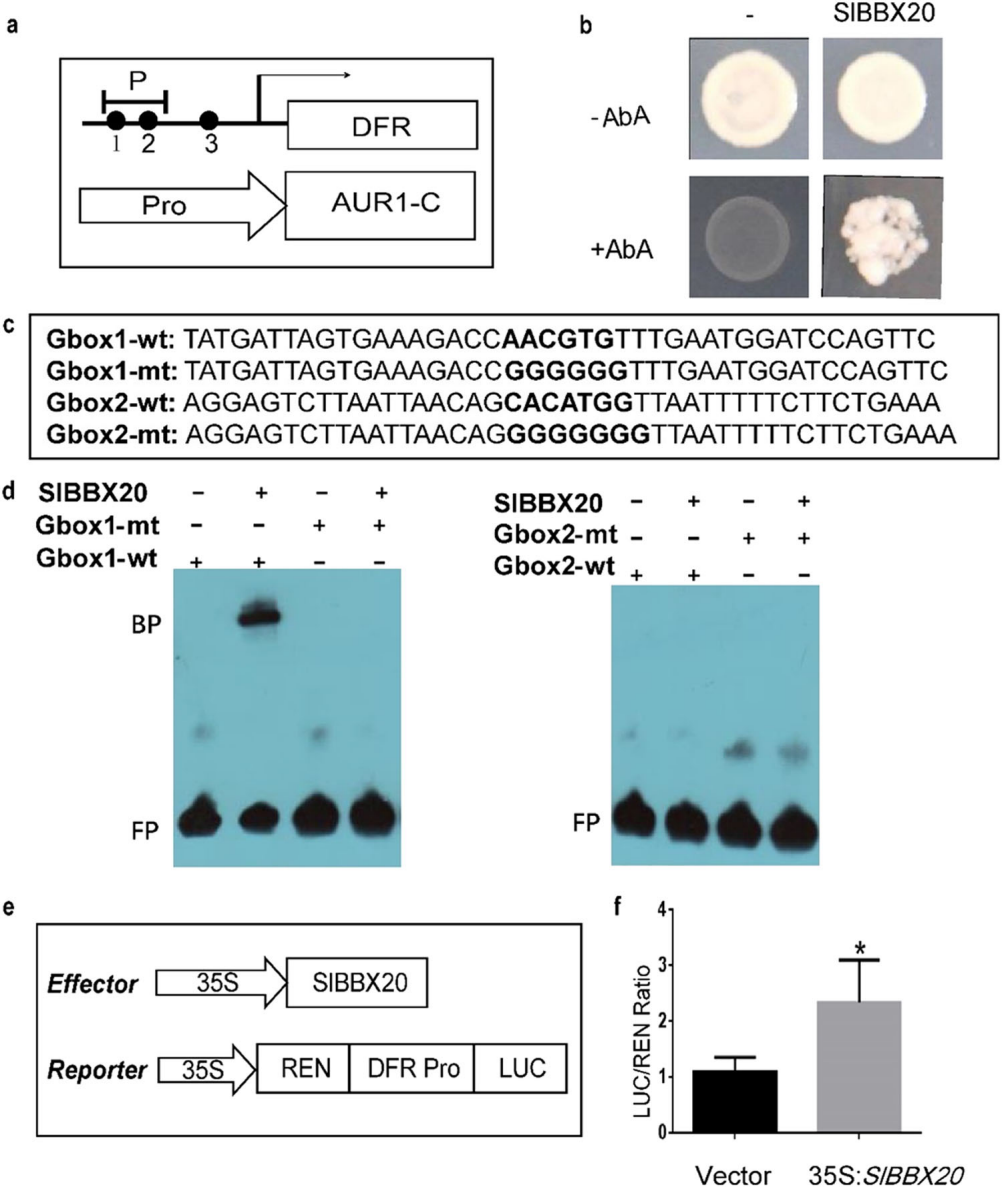
SlBBX20 activates the expression of *DFR* by binding its promoter. **a** Schematic diagram of the G-box locations on the *DFR* promoter. P1 includes G-box1 and G-box2, and the black dots indicate the locations of the G-boxes. **b** SlBBX20 was shown to bind the *DFR* promoter in a yeast one-hybrid assay. The yeast strain Y1HGold was transformed with the bait vector *pAbAi-DFR* and the prey vector *AD-SlBBX20* and plated on SD -Leu-Ura medium with or without aureobasidin A (45 ng mL^−1^). **c** Wild-type and mutant probe sequences used for EMSAs. G-box1-wt and G-box2-wt were used as wild-type probes. The synthesized G-box1-mt and G-box2-mt sequences were mutation probes in which the *cis*-element sequences were replaced by GGGGGG or GGGGGGG, respectively. **d** Affinity of SlBBX20 for two G-box motifs. BP indicates the binding probe; FP indicates the free probe. “+“ and “-“ indicate presence or absence in the EMSA, respectively. **e** Schematic of the vector used for the dual-luciferase experiment. **f** LUC/REN ratios. Luciferase activity was detected by dual-luciferase reporter assay

We hypothesized that SlBBX20 directly regulates transcription of the anthocyanin biosynthesis genes that were upregulated in the *SlBBX20*-overexpressing lines. BBX proteins were reported to regulate their target genes by binding G-boxes in their promoters. The *cis*-elements in the promoters of *DFR, ANS, CHS1, CHS2, F3H, F3'5’H,* and *FLS* were analyzed by using the PlantCARE database (http://bioinformatics.psb.ugent.be/webtools/plantcare/html/)^36^. The promoter of *ANS* does not contain a G-box. Therefore, we speculated that SlBBX20 cannot bind the *ANS* promoter. Yeast one-hybrid assays were conducted to test whether SlBBX20 regulates the other genes in this group. The yeast strain Y1GOLD was cotransformed with *AD-SlBBX20* and *pAbAi-CHS1*, *pAbAi-CHS2*, *pAbAi-F3H*, *pAbAi-F3'5’H*, *pAbAi-FLS* or a negative control. None of these transformants survived on the selective medium, which lacked Leu and Ura and contained AbA. These data indicate that SlBBX20 could not interact with the promoters of *CHS1, CHS2, F3H, F3'5’H,* or *FLS*, although these promoters contain G-boxes (data not shown). The promoter of *SlDFR* contains three G-boxes (Fig. 4a). We designed the G-box1, 2 and 3 sequences to confirm their interaction. We found that Y1GOLD yeast cells cotransformed with *AD-SlBBX20* and *pAbAi-SlDFR* (including G-box1, 2) could survive on the selective medium. However, *pAbAi-SlDFR* including G-box3 could not survive on the medium (data not shown). These data indicate that SlBBX20 can bind G-box1 or 2 in the promoter of *SlDFR* (Fig. 4b).

Next, we performed an EMSA to determine which G-box (G-box1 or 2) is targeted by SlBBX20. In the EMSAs, the SlBBX20 protein bound the G-box1-wt probe but not the other probes. This result demonstrated that SlBBX20 binds G-box1 in the *SlDFR* promoter in vitro (Fig. 4c, d). Subsequently, a dual-luciferase system assay was performed to test whether SlBBX20 could activate the expression of *SlDFR*. As shown in Fig. 4f, the ratio of LUC to REN in tobacco leaves co-injected with *62SK-SlBBX20* and *pAbAi-SlDFR* was increased by 2.6-fold relative to the negative control (*62SK* and *pAbAi-SlDFR*). These results provide evidence that SlBBX20 can activate the transcription of *SlDFR* by binding the G-box1 *cis*-element in its promoter.

### SlBBX20 interacts with SlCSN5-2 in vivo

**Fig. 5:**
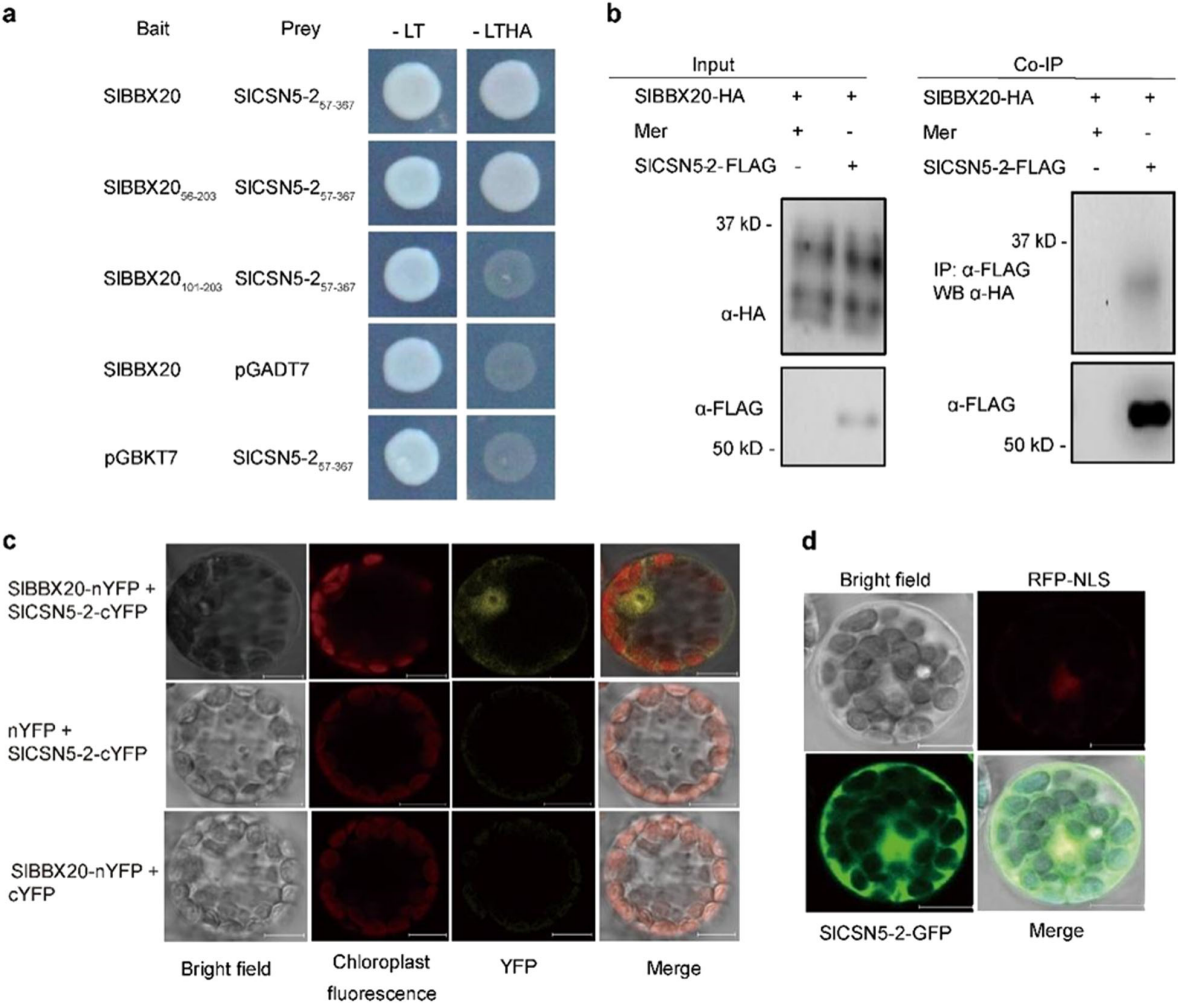
Interactions between SlBBX20 and SlCSN5-2. **a** Interaction between SlBBX20 and SlCSN5-2 in yeast two-hybrid experiments. The *BD-S1BBX20* and *AD-S1CSN5-2*_*57-367*_ (residues 57 to 367) plasmids were cotransformed into yeast strain AH109. **b** Interaction between SlBBX20 and SlCSN5-2 in coimmunoprecipitation assays. *SlBBX20-HA* and *SlCSN5-2-FLAG* were simultaneously introduced into tobacco protoplasts. The proteins were immunoprecipitated using anti-FLAG matrix beads and analyzed by western blotting. **c** Interaction between SlBBX20 and SlCSN5-2 in BiFC assays. The *pHBT-SlBBX20-nYFP* and *pHBT-SlCSN5-2-cYFP* plasmids were cotransformed into *Arabidopsis* protoplasts. The *pHBT-SlBBX20-nYFP* and *cYFP* plasmids or *pHBT-SlCSN5-2-cYFP* and *nYFP* plasmids as a negative control were used. YFP fluorescence was observed using confocal laser scanning microscopy. **d** Subcellular localization of SlCSN5-2. *SlCSN5-2* without a stop codon was cloned into *pHBT-GFP*. Tobacco protoplasts were extracted and cotransformed with plasmids encoding SlCSN5-2-GFP and a nuclear marker fused to RFP

To further analyze the molecular mechanism by which SlBBX20 regulates anthocyanin content, a yeast two-hybrid (Y2H) screen was performed using SlBBX20 as bait. We found that the protein encoded by Solyc06g073150 could interact with SlBBX20 in the Y2H screen. The full-length coding sequence of this gene is 1104 bp in length. The gene encodes a predicted protein consisting of 367 amino acid (aa) residues, which was referred to as SlCSN5-2 in a previous study^37^. We used three different methods to confirm the interaction between the SlBBX20 and SlCSN5-2 proteins. First, the interaction was verified using the Y2H assay (Fig. 5a). The full-length SlCSN5-2 protein was previously demonstrated to generate false-positive results in the Y2H assay. Therefore, a truncated *SlCSN5-2* construct (*SlCSN5-2*_*57-367*_) was used to test the interaction. SlBBX20 was also divided into two fragments consisting of residues 56-203 or 101-203. The yeast cells cotransformed with the *BD-SlBBX20/BD-SlBBX20*_*56-203*_ and *AD-SlCSN5-2*_*57-367*_ plasmids grew on SD -Leu/Trp/His/Ade medium, but the negative control yeast cells and yeast cells cotransformed with *BD-SlBBX20*_*101-203*_ and *AD-SlCSN5-2*_*57-367*_ did not grow on this medium, suggesting an interaction between *SlBBX20*_*56-101*_ and *SlCSN5-2*_*57-367*_.

Second, we employed a coimmunoprecipitation assay to confirm the interaction (Fig. 5b). *SlBBX20-HA* and *SlCSN5-2-FLAG* plasmids were cotransformed into tobacco protoplasts and expressed for 8–10 h. The proteins were immunoprecipitated using an anti-FLAG antibody and immunoblotted using an anti-HA antibody. The SlBBX20-HA protein was coimmunoprecipitated with SlCSN5-2-FLAG, but the negative control was not (Fig. 5b).

Finally, the interaction between SlBBX20 and SlCSN5-2 was tested using the bimolecular fluorescence complementation (BiFC) assay. We observed YFP fluorescence in tobacco protoplast cells cotransformed with the *SlBBX20-nYFP* and *SlCSN5-2-cYFP* plasmids but not in the negative control tobacco protoplast cells cotransformed with the empty vector and *SlBBX20-nYFP* or *SlCSN5-2-cYFP* plasmids (Fig. 5c). These data provide in vivo evidence that SlBBX20 interacts with SlCSN5-2.

We also determined the subcellular localization of SlCSN5-2, and the results showed that SlCSN5-2 accumulates in both the nucleus and cytoplasm (Fig. 5d). This localization pattern was very similar to that of SlBBX20^34^, suggesting that SlCSN5-2 and SlBBX20 work together.

### *SlCSN5* regulates anthocyanin accumulation

**Fig. 6:**
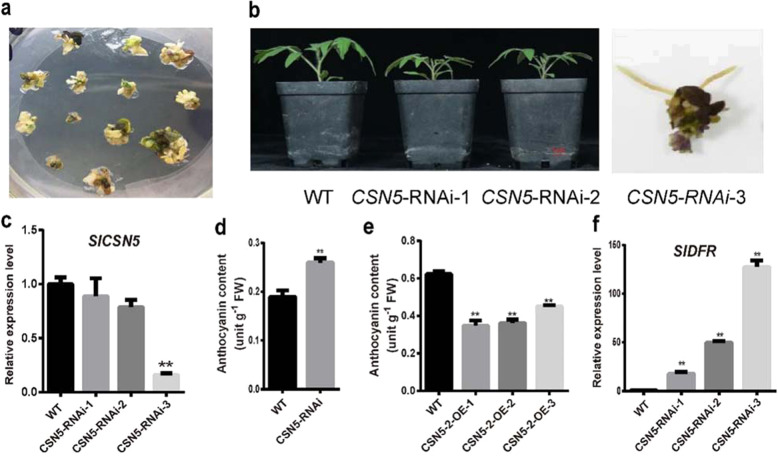
*SlCSN5* regulates anthocyanin accumulation. Calli (**a**) and seedlings (**b**) after *SlCSN5* interference. **c** Detection of *SlCSN5* expression in the *SlCSN5*-RNAi seedlings. **d** Anthocyanin content in the *SlCSN5*-RNAi plants. **e** Anthocyanin content in the *SlCSN5-2*-overexpressing plants. **f** The relative expression level of *SlDFR* in the *SlCSN5*-RNAi seedlings. Data represent the mean ± SE (*n* = 3). One-way ANOVA was performed. “*” and “**” indicate statistically significant differences with *P* < 0.05 and *P* < 0.01, respectively

To study the effects of *SlCSN5-2* on anthocyanin biosynthesis, we used RNA interference to downregulate the expression of *SlCSN5* (including *SlCSN5-1* and *SlCSN5-2*) in stably transformed plants. It was impossible to independently silence *SlCSN5-1* and *SlCSN5-2* in tomato due to their high sequence similarity^37^. While generating the transgenic plants, we observed many purple calli and shoots (Fig. 6a), similar to the effects of *SlBBX20* overexpression. Strongly *SlCSN5*-silenced calli accumulated many anthocyanins but failed to grow into normal plants. The moderately silenced plants survived, but their growth was significantly restrained (Fig. 6b, c), indicating that the function of *SlCSN5-2* in growth and development is indispensable. We selected a line in which *SlCSN5* expression was moderately decreased to test the anthocyanin content and found that it was significantly increased compared with that in the WT plants (Fig. 6d). Correspondingly, when *SlCSN5-2* was overexpressed in tomato, the anthocyanin content was significantly decreased compared with that in the WT plants (Fig. 6e), suggesting that *SlCSN5* is a negative regulator of anthocyanin biosynthesis.

Furthermore, to confirm whether *SlCSN5-2* regulates the accumulation of anthocyanin by *DFR*, we detected the expression level of *SlDFR* in *SlCSN5*-RNAi plants. The expression level of *SlDFR* was significantly upregulated compared with that of the WT plants, and the expression level of *SlCSN5-2* was negatively correlated with that of *SlDFR* (Fig. 6f). This result suggests that *SlCSN5* negatively regulates the accumulation of anthocyanin in tomato by *SlDFR*.

### *SlCSN5-2* regulates accumulation of the SlBBX20 protein

**Fig. 7:**
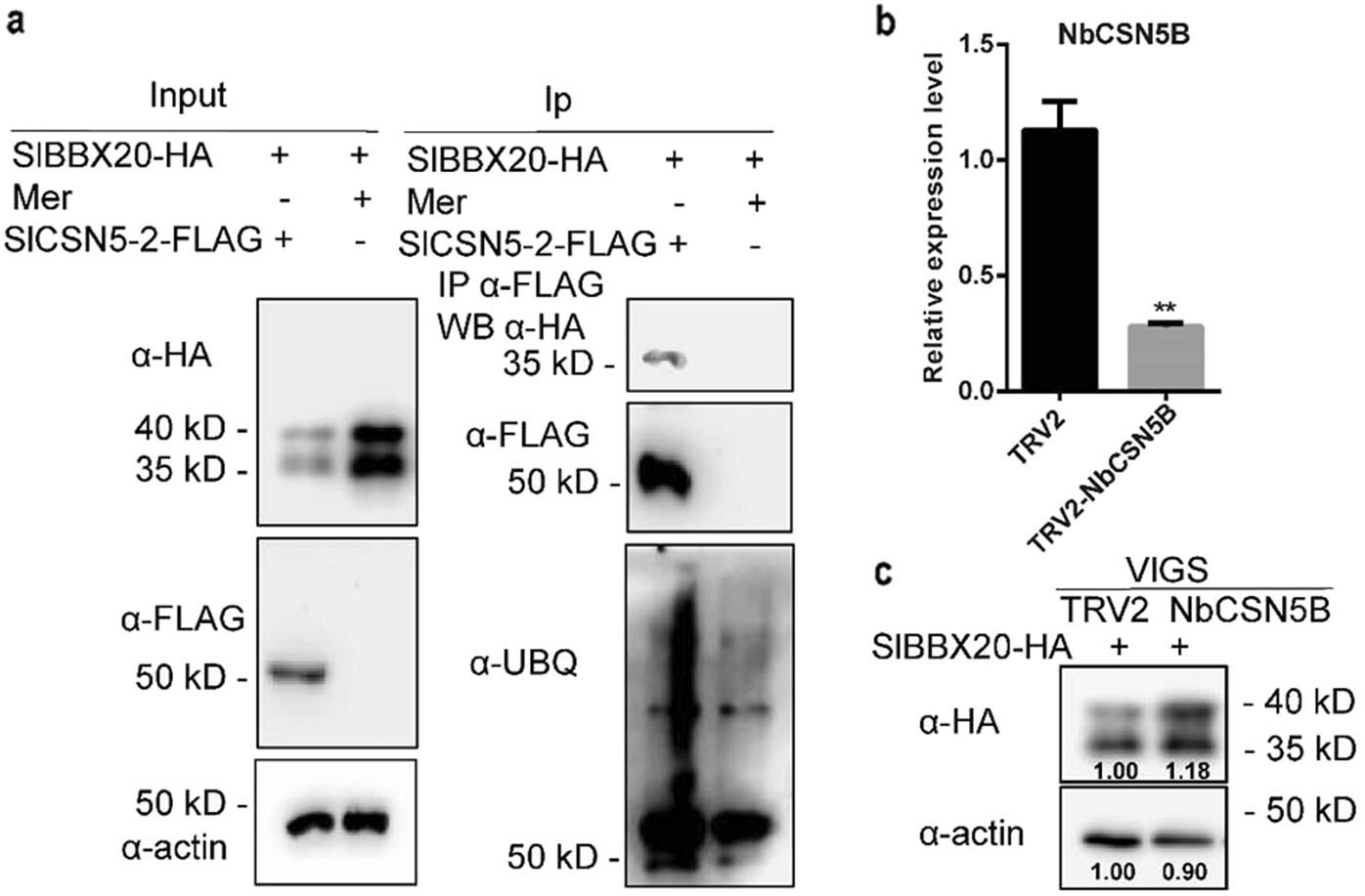
SlCSN5-2 regulates accumulation of the SlBBX20 protein. **a** The effect of SlCSN5-2 on ubiquitination of the SlBBX20 protein. The *SlBBX20-HA* and *SlCSN5-2-FLAG* plasmids were cotransformed into tobacco protoplasts. Anti-FLAG matrix beads were used for immunoprecipitation experiments. Finally, several antibodies (anti-HA, anti-FLAG, and anti-UBQ) were used to detect the accumulation of different proteins, and actin was used as a control. **b** Relative *NbCSN5B* expression level in *NbCSN5B*-VIGS plants. Tobacco leaves were infiltrated with *pTRV2-NbCSN5B* and *pTRV2* as controls. **c** *NbCSN5B* downregulated the SlBBX20-HA protein. *NbCSN5B* was silenced in tobacco by virus-induced gene silencing. The protoplasts were extracted and assessed using anti-HA, TRV2 as a control

Previously, we revealed that SlBBX20 is modulated by the E3 ubiquitin ligase CRL4^34^. SlCSN5-2 was reported to regulate CRL ubiquitin ligase and may regulate ubiquitination of the SlBBX20 protein by modifying the E3 ubiquitin ligase CRL4. To explore whether SlCSN5-2 affects ubiquitination of the SlBBX20 protein, we conducted an immunoprecipitation assay to detect the ubiquitination of SlBBX20 with an anti-UBQ antibody. The results showed that upon SlCSN5-2 coexpression, ubiquitination of the SlBBX20 protein was enhanced, and expression of the SlBBX20 protein decreased with actin used as an input control (Fig. 7a).

*NbCSN5B* in tobacco is an ortholog of *SlCSN5-2*, and their gene sequences are highly similar (Supplementary Fig. S1). We used VIGS to silence *NbCSN5B* in tobacco. Relative *NbCSN5B* gene expression was confirmed to be downregulated in leaves (Fig. 7b). Then, we extracted protoplasts from tobacco and transiently expressed SlBBX20-HA in the protoplasts. After 10 h, the proteins were extracted and detected by western blotting (Fig. 7c). We found that the SlBBX20-HA protein accumulated to higher levels in the *NbCSN5B*-silenced plants than in the control plants. In general, these results suggest that SlCSN5-2 interacts with SlBBX20 and promotes its ubiquitination and degradation to negatively regulate anthocyanin biosynthesis.

## Discussion

Anthocyanins are natural plant pigments involved in regulating the coloration of specific plant organs, such as leaves, flowers, and fruits. Previous studies on the regulation of anthocyanin biosynthesis have mainly focused on the MBW complex. Some positive regulators of anthocyanin biosynthesis, such as SlAN2, SlAN1, and SlMYB75^14,15^, and some negative regulators of anthocyanin biosynthesis, such as MdMYB16, FaMYB1, and PhMYB27^20,38,39^, have been reported in different plants. Recently, some BBX proteins in apple (i.e., MdCOL4, MdBBX20, MdBBX22) were found to regulate anthocyanin biosynthesis^2–4,33^. In pear, PpBBX16, PpBBX18, PpBBX21, and PpBBX24 were reported to be involved in anthocyanin synthesis^5,6,32^. A recent study showed that MdBBX37 inhibits the transactivating activities of MdMYB1 and MdMYB9 and therefore downregulates anthocyanin biosynthesis^3^.

In this study, we found that SlBBX20 interacts with SlCSN5-2 and promotes anthocyanin biosynthesis by binding the *DFR* promoter. Tomato plants overexpressing *SlBBX20* accumulated high levels of anthocyanins. Anthocyanins are synthesized by the flavonoid pathway, which contains genes that contribute to anthocyanin biosynthesis at both the early and late stages^40^. We found that most of these genes were upregulated in the *SlBBX20*-overexpressing lines, although the extent to which they were upregulated varied. BBX transcription factors were reported to regulate transcription by binding G-box *cis*-elements^4,6,33^. Here, SlBBX20 was found to directly bind the first G-box in the *SlDFR* promoter and activate its expression, and the accumulation of anthocyanins was highly correlated with the expression level of *SlBBX20*. DFR is a key enzyme in the anthocyanin synthesis pathway, its mutation blocks the accumulation of anthocyanin in tobacco, and a white flower phenotype appeared^41^. MdBBX20 was reported to promote anthocyanin biosynthesis by binding the promoters of *DFR*, *ANS,* and *MYB1*^2^. PpBBX16 requires PpHY5 to increase the expression levels of genes related to anthocyanin biosynthesis^5^. Thus, the mechanisms used by BBX20 to promote anthocyanin biosynthesis appear to be similar but vary among different plant species.

A large number of studies in *Arabidopsis* have shown that BBX family proteins are involved in photomorphogenesis^26–31^. Our previous study showed that *SlBBX20*-overexpressing tomato plants exhibited enhanced photomorphogenesis^34^. We identified the SlCSN5-2 protein as a binding partner of SlBBX20. The COP9 signalosome (CSN) plays an important role in plant photomorphogenesis and was originally discovered by cloning mutant alleles that disrupt photomorphogenesis in *Arabidopsis*^42^. The accumulation of anthocyanins is an important phenomenon in photomorphogenesis. Recently, several BBX proteins were found to interact with the HY5 protein and regulate anthocyanin accumulation in *Arabidopsis*, apple, and pear^3,6,35^. UV-B radiation induces the accumulation of anthocyanins using a signaling mechanism that depends on MdCOP1; This mechanism activates MdHY5 and promotes the binding of MdHY5 to the *MdMYB* gene-promoter region^7^. In this study, we found that CSN5-2—a photomorphogenesis factor—could negatively regulate the accumulation of anthocyanins by promoting the accumulation of the SlBBX20 protein. Although the *SlBBX2*0-OE plants accumulated many anthocyanins under light, anthocyanin accumulation in the *SlBBX20*-OE plants was reduced upon exposure to dark conditions for a period of time, and the expression of *SlDFR* was significantly downregulated compared with that under light (Supplementary Fig. S2a, b, d). suggested that the anthocyanin modulation by *SlBBX20* is dependent on light. The transcription level of *SlCSN5-2* was not significantly changed when the plants were exposed to dark conditions (Supplementary Fig. S2c). Previous studies have indicated that the protein level of COP9 is not affected by light, but light may regulate the activity of the SlCSN5-2 protein at the posttranslational level^43^. Therefore, we believe that light is required for anthocyanin regulation in the SlBBX20-SlCSN5-2 model. These data reveal a novel regulatory pathway involved in light-induced anthocyanin biosynthesis.

Interestingly, when we knocked down the expression of *SlCSN5* in tomato using RNAi, anthocyanins accumulated in the calli and shoots, and strongly silenced plants accumulated abundant anthocyanins but failed to grow into normal plants. A moderate decrease in the level of *SlCSN5* expression led to dwarfing, indicating the important function of *SlCSN5* in tomato development. Tomato contains two *CSN5* genes, namely, *SlCSN5-1* and *SlCSN5-2*. Because their sequences are highly similar, it is difficult to individually interfere with the expression of these genes. Indeed, previous studies also reported that it was impossible to use different sequences to separately silence the two *CSN5* genes in tomato^37^. *SlCSN5*-VIGS plants were reported to be approximately 50% shorter in stature than controls^37^. The effects of *SlCSN5* on growth and development may have been more severe in our stably transformed tomato lines. Among *Arabidopsis thaliana*, the *csn5a* mutant develops purple cotyledons^44^, but the reason is unknown. We found the sequence similarity between SlCSN5-2 and AtCSN5a to be 84%. We speculate that AtCSN5a utilizes a mechanism to regulate anthocyanin biosynthesis that is similar to that of SlCSN5-2.

CSN can regulate protein degradation through the ubiquitination pathway. The major activity of CSN is regulated by the fifth subunit (*CSN5*)^45^. CSN5 has been reported to be involved in deneddylation activity^46^. It can regulate the activity of CRLs (Cullin RING ligases) by covalently binding and removing RUB proteins^47,48^. Ubiquitinated proteins have been reported to accumulate in *Arabidopsis csn* mutants^49^. In the present study, we found that the ubiquitination of SlBBX20 was enhanced when it was coexpressed with SlCSN5. Furthermore, when we silenced the *SlCSN5* homolog *NbCSN5B* in tobacco, accumulation of the SlBBX20 protein increased, indicating that CSN5 regulates accumulation of the SlBBX20 protein. Therefore, we infer that CSN5 is involved in ubiquitination and degradation of the SlBBX20 protein. Our previous study demonstrated that SlBBX20 can be ubiquitinated by the CUL4-DET1-DDB1 complex and eventually degraded by the 26 S proteasome^34^. CSN was found to modify the activity of several CUL4-based E3 ubiquitin ligases to regulate plant photomorphogenesis^50^. CSN5 might participate in regulating the activity of CUL4-based E3 ubiquitin ligases^51^. However, how CSN5 regulates the activity of CRLs and the accumulation of substrate remains unknown. Our work might provide insight into the modification of CSN5 to CRLs.

## Materials and methods

### Plant materials

The *SlBBX20* gene was cloned into the overexpression vectors *pHellsgate8* and *pCAMBIA2300-HA*. Using the “Alisa Craig” (LA2838A) tomato as the wild-type background, transgenic tomato plants were obtained by *Agrobacterium tumefaciens*-mediated transformation. The expression of *SlBBX20* in the transgenic plants was quantified using qRT-PCR.

### Gene expression analysis

TRIzol reagent (Invitrogen, USA) was used to extract total RNA from leaves as previously described^52^. cDNA was synthesized using a HiScript^®^ II 1st Strand cDNA Synthesis Kit (Vazyme, China). Gene-specific oligonucleotides were used to perform qRT-PCR in a Roche LightCycler 480 system^53^. Relative gene expression was calculated by Microsoft Excel. Expression of the *actin* gene (SGN-U580609) was used as an internal control. The sequences of the gene-specific oligonucleotides used in the analysis are listed in Supplementary Table S2.

### Measurement of the total anthocyanin content

The methanol-HCl method was used to extract anthocyanins from tomato leaves. Approximately 2 g of tomato leaves ground with liquid nitrogen was soaked in 5 ml of 1% (v/v) methanol HCl and extracted overnight in the dark at 24 °C. A spectrophotometer (UV-1600, Shimadzu, Japan) was used to measure the absorbance of each sample at 530, 620, and 650 nm. The following formula was used to calculate the relative anthocyanin content: optical density (OD) = (OD_530_-OD_620_)-0.1(OD_650_-OD_620_). One unit of anthocyanin represented a change in the OD of 0.1.

### RNA sequencing

Three biological replicates from 4-week-old tomato seedlings that overexpressed *SlBBX20* and WT tomato seedlings were selected for the extraction of total RNA and RNA sequencing. We used the average RPKM (reads per kilobase per million reads) value as a measure of gene expression^54^. Genes showing at least a twofold change in expression with an FDR-adjusted p-value of less than 0.05 were defined as differentially expressed genes (DEGs). The heat map was plotted with log_2_RPKM values to visually show differences in expression levels.

### Yeast one-hybrid assay

The full-length *SlBBX20* gene was amplified with tomato cDNA as the template and inserted into *pGADT7* to obtain the prey vector (*AD-SlBBX20*). Fragments of the *DFR, CHS1, CHS2, F3H, F3'5’H,* and *FLS* promoters were amplified with tomato genomic DNA as the template and cloned into *pAbAi* to obtain a bait vector (*pAbAi-DFR*, *CHS1*, *CHS2*, *F3H*, *F3'5’H,* and *FLS*). The yeast strain Y1HGold transformed with bait vector was cultured on SD -Ura medium and placed in a 30 °C incubator for three days. Subsequently, the prey vector was transformed into the yeast strain Y1HGold previously transformed with the bait vector, and the resulting strain was plated on SD -Leu-Ura medium. The positive clones were diluted with 0.9% NaCl to an OD600 of 0.1, after which 2 μL of each suspension was spotted on SD -Leu medium with or without aureobasidin A (45 ng mL^−1^).

### Transient dual-luciferase assay

The full-length *SlBBX20* ORF was amplified by using tomato cDNA as a template and inserted into the *pGreen II 62-SK* vector. Promoter fragments from *DFR* (bp 1 to 1490) were cloned into the reporter vector, *pGreen II 0800-LUC*. The constructed vectors were individually introduced into *Agrobacterium* strain GV2260. The *Agrobacterium* liquid introduced into the reporter vector and effect vector were mixed and injected into tobacco leaves. Transient expression was evaluated three days after infiltration^55^. The firefly luciferase activity was detected by a dual-luciferase reporter assay system (Promega, USA).

### Electrophoretic mobility shift assays (EMSAs)

The *SlBBX20* gene was cloned into *pET28a* to express the His-tagged SlBBX20 protein. Based on the *DFR* promoter sequence, distinct 30-bp single-stranded fragments containing the *cis*-acting elements AACGTG or CACATGG were synthesized (TsingKe, China) and labeled using the Biotin 3′ End DNA Labeling Kit (Thermo Scientific, USA). The *cis*-acting element was replaced with a series of guanosines to obtain the mutated fragment. The labeled DNA fragment and purified His-SlBBX20 protein were incubated in the reaction mixture for 30 min as described previously^18^. The protein-DNA complexes were separated in 6.5% native PAGE gels. The gels were transferred to a nylon membrane (Beyotime Biotechnology, China). After UV cross-linking, chemiluminescence was used to observe the migration of the biotin-labeled probe on the membranes.

### Yeast two-hybrid assay

The *SlBBX20* coding sequence was inserted into the prey vector *pGBKT7* (BD) to yield *BD-SlBBX20*, which was used as bait to screen a tomato yeast two-hybrid library, which showed that SlCSN5-2 and SlBBX20 interact. Furthermore, the coding sequence for a truncated SlCSN5-2 protein construct lacking 56 amino acids at its N-terminus (residues 57 to 367, SlCSN5-2_57-367_) was amplified and cloned into the bait vector *pGADT7* (AD) to yield *AD-SlCSN5-2*. The *BD-S1BBX20* and *AD-S1CSN5-2* plasmids were cotransformed into yeast strain AH109. After transformation, yeast AH109 cells were grown on SD -Trp-Leu medium for 3 days. A single clone was spotted in SD -Trp-Leu-His-Ade medium, and the growth of yeast cells was observed. The *pGBKT7* and *AD-SlCSN5-2* plasmids or *BD-SlBBX20* and *pGADT7* plasmids were used as negative controls.

### Bimolecular fluorescence complementation (BiFC)

The *SlBBX20* coding sequence was inserted into *pHBT-nYFP* to yield *pHBT-SlBBX20-nYFP*. The full-length *SlCSN5-2* coding sequence was cloned into *pHBT-cYFP* to yield *pHBT-SlCSN5-2-cYFP*. Two plasmids, *pHBT-S1BBX20-nYFP* and *pHBT-S1CSN5-2-cYFP*, were cotransformed into *Arabidopsis* protoplasts. After the protoplasts had been cultured for 12 h, YFP fluorescence was observed by confocal microscopy.

### Coimmunoprecipitation

For coimmunoprecipitation assays, tobacco protoplasts coexpressing *SlBBX20-HA* and *SlCSN5-2-FLAG* or expressing *Mer* as a control were collected, resuspended in extraction buffer and centrifuged at 12000 × g at 4 °C for 10 min. Five microliters of anti-FLAG matrix beads (Sigma, USA) were added to the supernatant and incubated at 4 °C for 2 h to capture the epitope-tagged protein. Finally, anti-HA (MBL, Japan) or anti-FLAG (Sigma, USA) antibodies were used for western blot analysis. Anti-UBQ (Millipore, USA) was used to detect ubiquitination of the SlBBX20 protein, and anti-actin was used as a control.

### Subcellular localization

The *SlCSN5-2* coding sequence without a stop codon was amplified and cloned into *pHBT-GFP* using gene-specific primers (Supplementary Tables S1). Tobacco protoplasts were extracted and cotransformed with the *SlCSN5-2-GFP* plasmid and a nuclear marker fused to RFP. After expression in protoplasts for 10 h, fluorescence was observed under a laser scanning confocal microscope.

### Downregulation of CSN5 expression by virus-induced gene silencing (VIGS)

Because the two CSN5 sequences in tomato (CSN5-1 and CSN5-2) and two CSN5B sequences in tobacco (CSN5B-1 and CSN5B-2) are highly homologous, it is difficult to individually interfere with the two CSN5 sequences in a single species. Therefore, we elected to interfere with the expression of both. We employed virus-induced gene silencing (VIGS) and stable RNA interference (RNAi) to downregulate the expression of *CSN5* in tobacco and tomato, respectively. For VIGS, we selected a unique sequence from *NbCSN5B* and ligated the sequence into *pTRV2*. The recombinant plasmid was transferred into *Agrobacterium* GV3101. *Agrobacterium* strains transformed with *NbCSN5B-TRV2* and *TRV1* or *TRV2* and *TRV1* were mixed and injected into tobacco leaves. After 10 days, the protoplasts were extracted from the tobacco, and *SlBBX20-HA* was transiently expressed. After SlBBX20-HA had been expressed in the protoplasts for 10 h, SlBBX20-HA was extracted and detected by western blotting. A unique sequence was selected from *SlCSN5* and used to construct an RNAi vector for stable transformation. The sequences of the gene-specific oligonucleotides used in the analysis are listed in Supplementary Table S1.

### Statistical analysis

Statistical analyses were performed using Prism 6 and SPSS 26.0. All of the experiments were repeated at least three times. Statistically significant differences were determined by subjecting the data to one-way ANOVA. The data are reported as the mean value ± SE. * indicates *P* < 0.05, and ** indicates *P* < 0.01.

## Supplementary information

Supplementary file
