# Supplementary material for: SlBBX20 interacts with the COP9 signalosome subunit SlCSN5-2 to regulate anthocyanin biosynthesis by activating SlDFR expression in tomato
Source: Hortic Res. 2021 Jul 1;8:163. doi: 10.1038/s41438-021-00595-y (PMC8245592; doi:10.1038/s41438-021-00595-y)
Supplement: Supplementary file 1 — Supplementary file [file 41438_2021_595_MOESM1_ESM.docx]

**Supplementary Data**

**Table S1.** List of primers used in this study.

| Primer name | Primer sequence 5’-3’ |
| --- | --- |
| AD-SlBBX20-Fw | GTACCAGATTACGCTCATATGATGAAGATTCAATGTGATGTTTGT |
| AD-SlBBX20-Rv | ATGCCCACCCGGGTGGAATTCTCAAAATTCATAGGAAGAAGAAGAG |
| pAbAi-DFR(F)-Fw | AGCTTGAATTCGAGCTCGGTACCTATGATTAGTGAAAGACCAACGTG |
| pAbAi-DFR(F)-Rv | ACATACAGAGCACATGCCTCGAGGCAATGCTTGTCGCAGTTT |
| pAbAi-DFR(N)-Fw | AGCTTGAATTCGAGCTCGGTACCAAACTGCGACAAGCATTGC |
| pAbAi-DFR(N)-Rv | ACATACAGAGCACATGCCTCGAGCAAATGTGACTGGTTGGTGAGA |
| PAbAi-F3H -Fw | AGCTTGAATTCGAGCTCGGTACCATCCGTAATGCGTATTATTAGATATG |
| pAbAi-F3H -Rv | ACATACAGAGCACATGCCTCGAGATTACTACCCTTCGTGCCAAC |
| PAbAi-CHS2-Fw | AGCTTGAATTCGAGCTCGGTACCTAACTCCCTCCATATCTCCTTGA |
| pAbAi- CHS2-Rv | ACATACAGAGCACATGCCTCGAGGGAGTCGCCGTGCCTAT |
| PAbAi-FLS-Fw | AGCTTGAATTCGAGCTCGGTACCGAAACACGCCACAAACGG |
| pAbAi- FLS-Rv | ACATACAGAGCACATGCCTCGAGATTGCTTGGACCCTTGCTAC |
| PAbAi-AN2-Fw | AGCTTGAATTCGAGCTCGGTACCAAACTTTCCAATGATACCGTAGAG |
| pAbAi- AN2-Rv | ACATACAGAGCACATGCCTCGAGGATGCCACTTTCCTTCACCA |
| PAbAi-F35H-Fw | AGCTTGAATTCGAGCTCGGTACCCGAAATACTATCCCAAAGAAGTAAC |
| pAbAi- F35H-Rv | ACATACAGAGCACATGCCTCGAGATAGCCGCAACAAATAACTCAT |
| PAbAi-UFGT-F | AGCTTGAATTCGAGCTCGGTACCGAAAGGAGGGCATAACCGTA |
| pAbAi- UFGT-R | ACATACAGAGCACATGCCTCGAGCGCCACCTTCATCACGAC |
| BD-SlBBX20-Fw | TCAGAGGAGGACCTGCATATGATGAAGATTCAATGTGATGTTTGT |
| BD-SlBBX20-Rv | TCGACGGATCCCCGGGAATTCTCAAAATTCATAGGAAGAAGAAGAG |
| AD-SlCSN5-2_57-367_-Fw | GTACCAGATTACGCTCATATGGAGAAGCCGTGGGCGAG |
| AD-SlCSN5-2_57-367_-Rv | ATGCCCACCCGGGTGGAATTCTCAGCTTTCGATCATGGGC |
| SK-SlBBX20-Fw | GCCGCTCTAGAACTAGTGGATCCATGAAGATTCAATGTGATGTTTGT |
| SK-SlBBX20-Rv | TTGGTACCGGGCCCCCCCTCGAGTCAAAATTCATAGGAAGAAGAAGAG |
| 0800-DFR-Fw | CACTATAGGGCGAATTGGGTACCTATGATTAGTGAAAGACCAACGTG |
| 0800-DFR-Rv | TATGTTTTTGGCGTCTTCCATGGTTTCAGAAATGAAAGGTAAAAAAG |
| TRV-NbCSN5B-Fw | CTGTGAGTAAGGTTACCGAATTCTACAAGCCTCCAGATGATCCCA |
| TRV-NbCSN5B-Rv | CGCGTGAGCTCGGTACCGGATCCCGACAGTAATCTTAGCACTATCAC |
| RNAi-SlCSN5-2-Fw | GGGGACAAGTTTGTACAAAAAAGCAGGCTAGAAGCCGTGGACGAGTG |
| RNAi-SlCSN5-2-Rv | GGGGACCACTTTGTACAAGAAAGCTGGGTCGAAAGGCACCAATCTCAA |

Note: The horizontal line represents the restriction site

**Table S2.** qRT-PCR primer sequences used to quantify the expression genes.

| Primer name | Primer sequence 5’-3’ |
| --- | --- |
| SlCHS2-qPCR-Fw | GGCCGGCGATTCTAGATCA |
| SlCHS2-qPCR-Rv | TTTCGGGCTTTAGGCTCAGTT |
| SlCHS1-qPCR-Fw | CCCAATTATAGGGGTCGAAAGA |
| SlCHS1-qPCR-Rv | GGAACATCCTTGAGTAAGTGGA |
| SlF3H-qPCR-Fw | TGAAAAGACCCTTGAAACAA |
| SlF3H-qPCR-Rv | CGATTCTCTCACATATTTCA |
| SlFLS-qPCR-Fw | TAAGATTTGGCCTCCTCCTG |
| SlFLS-qPCR-Rv | ACCAAGCCCAAGTGATAAGC |
| SlF3'5'H-qPCR-Fw | CCACGTTGGAAGTTGCTAAG |
| SlF3'5'H-qPCR-Rv | TGGCTTGCATCGAACATC |
| SlDFR-qPCR-Fw | GAAGGCTGCAATGGAAGAAG |
| SlDFR-qPCR-Rv | GATTAAGCTTGGTGGGAACG |
| SlANS-qPCR-Fw | AGGGGTTTTAGGCCAGATG |
| SlANS-qPCR-Rv | ATGTCCAAGGCTATGGAAGC |
| SlActin-qPCR-Fw | TCTCTGTTGGCCTTGGGATT |
| SlActin-qPCR-Rv | CTTCGAGTTGCTCCTGAGGAA |
| NbCSN5B-qPCR-Fw | ACGATTGAGGTGATGGGACT |
| NbCSN5B-qPCR-Rv | CCGACCAGCCTGCTTGT |
| SlCSN5-2-qPCR-Fw | AAATTCAGATTGTAGCGCAGTG |
| SlCSN5-2-qPCR-Rv | ATGTTGTTCTCTAACTCCCAGG |


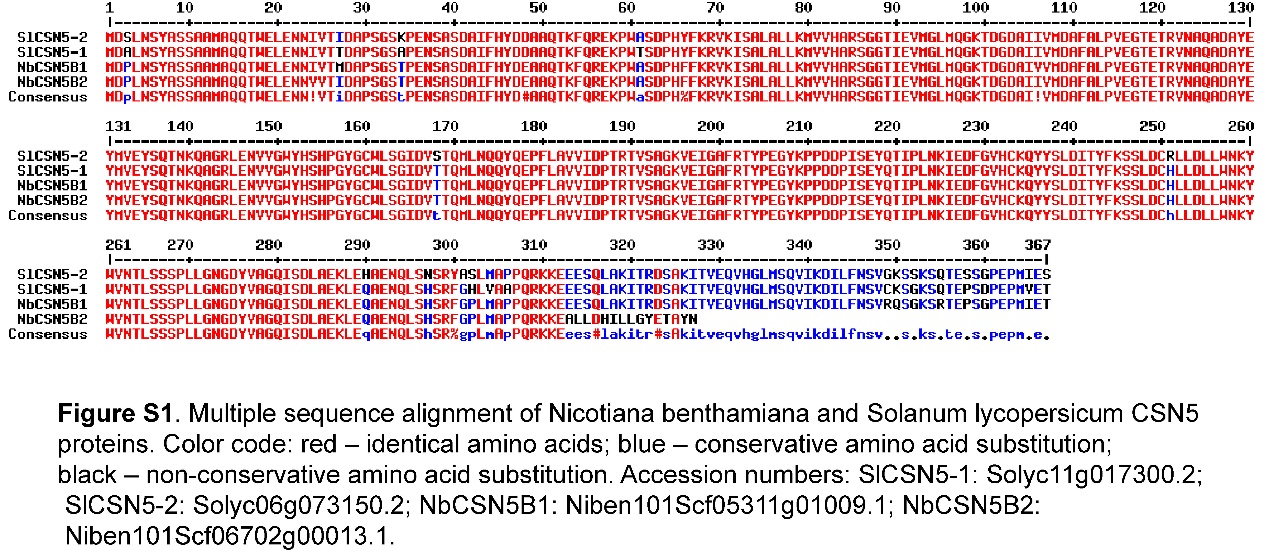


**Figure S1.** **Multiple sequence alignment of *Nicotiana benthamiana* and *Solanum lycopersicum* CSN5 proteins.** Accession numbers: *SlCSN5-1*(Solyc11g017300.2), *SlCSN5-2*(Solyc06g073150.2), *NbCSN5B1* (Niben101Scf05311g01009.1) and *NbCSN5B2* (Niben101Scf06702g00013.1).


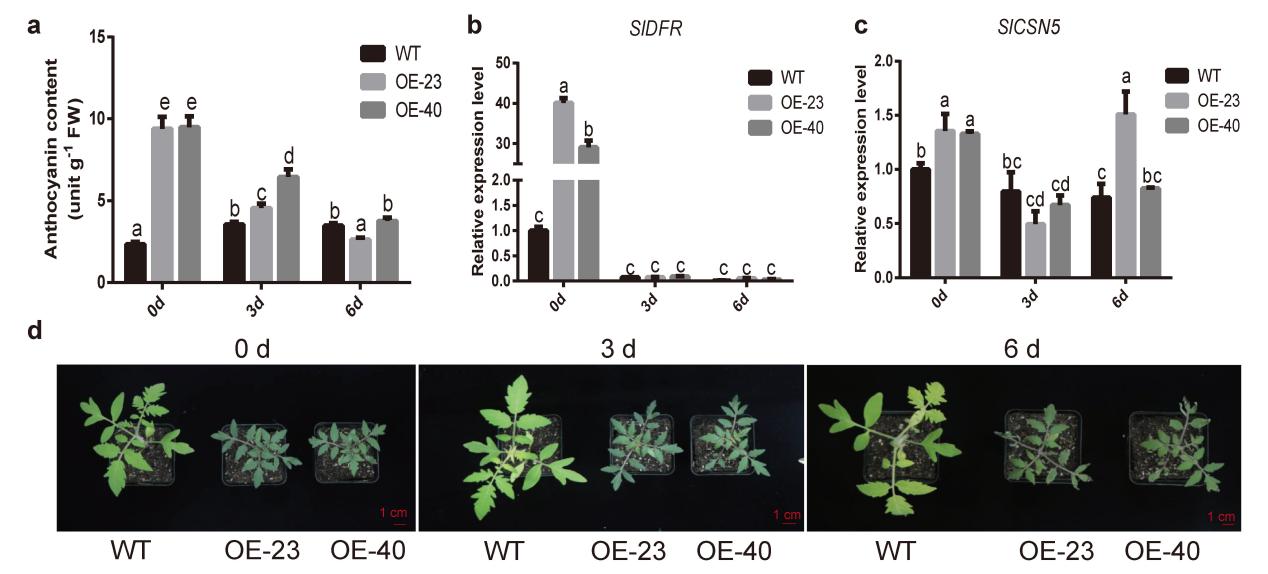


**Figure S2. Anthocyanin content of *SlBBX20*-OE plants decreases under dark conditions.** **a** Anthocyanin content of *SlBBX20*-OE plants at different times under dark. The relative expression levels of *SlDFR* (**b**) and *SlCSN5* (**c**) in *SlBBX20*-OE plants at different times under dark. **d** The photographs of *SlBBX20*-OE plants at 0 d, 3 d and 6 d under dark.
